# Healing Rates and Dressing Frequency of Silver Foam Dressings in Paediatric Burns: A Systemic Review and Meta-Analysis

**DOI:** 10.3390/ebj6010003

**Published:** 2025-01-27

**Authors:** Nathanael Q. E. Yap, Dilip K. Vankayalapati, Sum-Yu C. Lee, Hafsa O. Sulaiman, Alma Sato, M Zaid Shami, Valeria Antoniou, James W. F. Burns, Hayato Nakanishi, Christian A. Than, Graeme Southwick

**Affiliations:** 1 Addenbrookes, Cambridge University Hospital NHS Trust, Hills Rd., Cambridge CB2 0QQ, UK; 2 School of Medicine, St George’s University of London, London SW17 0RE, UK; 3 School of Medicine, University of Nicosia, Nicosia 2417, Cyprus; 4 Stoke Mandeville Hospital, Buckinghamshire NHS Trust, Oxford Thames Valley, Aylesbury HP21 8AL, UK; 5 Cleveland Clinic Foundation, Cleveland, OH 44195, USA; 6 Manchester Royal Infirmary, Manchester Foundation Trust, Manchester M13 9WL, UK; james.burns@mft.nhs.uk; 7 Department of Surgery, Mayo Clinic, Rochester, MN 55905, USA; 8 School of Biomedical Sciences, The University of Queensland, St Lucia, Brisbane, QLD 4072, Australia; 9 Melbourne Institute of Plastic Surgery, 253 Wattletree Road, Melbourne, VIC 3144, Australia

**Keywords:** paediatric, burns, silver, silver foam dressing, meta-analysis

## Abstract

Silver foam dressings have been extensively used in the management of burn injuries; however, its application in children requires elucidation. A literature search was conducted from database inception to October 2023. Eligible studies reported paediatrics patients under 21 years of age receiving silver dressings for burns or scalds. This review was registered prospectively with PROSPERO (CRD42023470239). 18 studies met the inclusion criteria with a total of 701 patients. The pooled mean time to re-epithelisation (TTRE) was 12.9 days (95% CI: 11.2, 14.6, I^2^ = 94%). The pooled mean duration of hospitalisation was 9.8 days (95% CI: 3.9, 15.7; I^2^ = 100%). Mean number of total dressing changes per patient was 3.6 (95% CI: 2.2, 4.9; I^2^ = 99%). There were a total of 98 complications, including 30 (30.6%) infections, 29 (29.6%) surgical requirements, 14 (14.3%) hypertrophic scarring, 17 (17.3%) escalations of care, 5 (5.1%) burn depth progression, and 3 (3.1%) others. Silver foam dressings appear as a safe and effective approach in terms of healing rate and dressing change frequency for selected paediatric patients with burn injuries. Despite the promising results, further comparative studies are required to evaluate the selection criteria and long-term effect of silver foam dressing.

## 1. Introduction

Burn injuries are a significant source of morbidity worldwide, causing both acute complications and lifelong physical and cosmetic impairments [1,2,3]. Children are at a disproportionately higher risk of burns due to their impulsiveness, risk naivety, and overall propensity for accidents [3,4,5]. Moreover, they are prone to deeper burns due to a thinner developing dermis, resulting in greater risks of infection as well as hypertrophic scarring and contractures [6,7]. Nonetheless, no consensus has been reached on the optimal approach to managing burn injuries in children.

Silver dressings have been used extensively in burn wound dressing due to their broad-spectrum antimicrobial coverage [8,9]. However, existing studies have suggested silver ions could impair healing through their dose-dependent cytotoxic effect on keratinocytes and fibroblasts [10]. The cytotoxic effect of silver ions has been demonstrated in vitro through cytotoxicity assays [11,12,13,14], yet the effect on healing has been hard to observe clinically [15]. Interestingly, some studies observed that silver dressings could promote wound healing independent of their antimicrobial effects, including their influence on neovascularisation and anti-inflammatory effects [11,16,17,18,19,20]. Nonetheless, two Cochrane reviews found insufficient evidence to support the use of silver dressings in improving healing rates [21,22].

Silver ions have a narrow therapeutic bactericidal concentration of 30–40 ppm with a short half-life in wounds [10]. Given that silver ions exert dose-dependent effects, frequent dressing changes are required to maintain the therapeutic concentration of silver. Frequent dressing changes in children could be painful, delay time to re-epithelisation (TTRE), and increase infective risk and hypertrophic scarring [23]. Emerging dressings contain silver in nanocrystalline states and in novel interfaces that function to maintain therapeutic concentrations for longer, thus theoretically reducing dressing frequency and risk of infection with improved healing [10,24,25].

Although silver foam dressings are established in the management of burn injuries, no consensus has been reached for the paediatric population. To our knowledge, no meta-analysis of existing studies has been conducted to evaluate the clinical feasibility of silver foam dressings in the context of selected burn injuries for paediatric patients. This meta-analysis aims to evaluate the safety and efficacy of silver foam dressings in paediatric populations for burn injuries with a primary focus on TTRE, duration of hospitalisation, frequency of dressing change, and complication rates, to provide a robust foundation for future advancements in paediatric burn care.

## 2. Materials and Methods

A comprehensive literature search was performed in multiple databases from inception to 12th October 2023, in accordance with Preferred Reporting Items for Systematic Reviews and Meta-analyses (PRISMA) guidelines [26]. The databases included PubMed, EMBASE (Elsevier), CiNAHL, Cochrane Central Register of Controlled Trials, Cochrane Database of Systematic Reviews, Scopus, and Web of Science. The search strategy from design was conducted by an experienced medical librarian together with the study’s principal investigator. The actual search strategy listed, all search terms used, and how they are combined are available in Appendix A. The review was registered prospectively with PROSPERO (CRD42023470239).

Eligible studies were randomised controlled trials (RCTs) or observational studies that met the following inclusion criteria: (1) Paediatric participants less than or equal to 21 years old; (2) Burn injuries treated with silver-containing foam dressing; (3) Reporting on at least one of the following primary outcomes: rate of burn healing, mean TTRE, or a number of dressing renewals. Case reports, case series, review articles, and abstracts were excluded from the study. This meta-analysis did not exclude studies based on sample size or language. Each article was independently screened twice by two of four blinded assessors (SCL, HOS, DKV, ZS). All conflicts were adjudicated by a fifth author (NQEY). The quality of each study was independently evaluated by two blinded assessors (NQEY and AS) using the Cochrane Collaboration Risk of Bias (ROB) 1 and ROB-2 Quality Assessment tool [27]. Any discrepancies were discussed by the two independent assessors, with disagreements addressed via an adjudicator (CAT).

The following data were extracted in this study: baseline (gender, age, weight), clinical (burn aetiology, depth, number, location, and total body surface area (TBSA)), and dressing characteristics (type, dressing frequency, silver formulation), as well as outcomes pertaining to healing (TTRE, method of determination of healing, duration of hospitalisation) and complications (infection, re-admission, and surgery requirement including skin grafting).

Paediatric inclusion criteria, age less than or equal to 21 years old, within this meta-analysis is in accordance with updated guidelines set by the American Academy of Paediatrics [28].

The healing endpoint for TTRE was defined as 95% burn epithelisation in three studies [29,30,31] and 100% in six studies [32,33,34,35,36,37]. In one article, the healing endpoint was characterised when dressings were no longer required as assessed by the attending surgeon [38]. TTRE was assessed visually, based on the experience and judgement of a burn surgeon or physician as reported in seven studies [29,30,32,34,35,37,38]. The method of assessment was not reported in three of the 10 studies that were analysed for TTRE [31,33,36].

In five studies, this duration was calculated from the time post-burn [29,32,33,35,38], while one study calculated this from the time following skin-graft transplantation [36]. The remaining four studies did not define whether healing duration was post-burn or post-dressing [30,31,34,37].

The pooled means and proportions of the data were analysed for single-arm meta-analysis using a random-effects, generic inverse variance method of DerSimonian and Laird, which assigns the weight of each study based on its variance [39]. The heterogeneity of effect size estimates across the studies was quantified using the Q statistic and I^2^ (*p* < 0.10 was considered significant). A value of I^2^ of 0–25% indicates insignificant statistical heterogeneity, 26–50% low heterogeneity, and 51–100% high heterogeneity [27]. Furthermore, a leave-one-out sensitivity analysis was conducted to assess each study’s influence on the pooled estimate by omitting one study at a time and recalculating the combined estimates for the remaining studies. Publication bias was assessed using a funnel plot [40]. If mean and standard deviation (SD) were unavailable, the median was converted to mean using the formulas from the Cochrane Handbook for Systematic Reviews of Interventions [20]. Data analysis was performed using Open Meta Analyst software (CEBM, Brown University, Providence, RI, USA).

## 3. Results

### 3.1. Study Selection

The initial literature search of the electronic database yielded 366 studies. After removing duplicates, the articles were screened for inclusion and exclusion criteria, and 82 studies were retained for full-text review. A total of 18 relevant studies with 701 participants met the criteria and were included in this meta-analysis. This included six RCTs [29,34,37,38,41,42], six prospective cohorts [31,35,36,43,44,45], and six retrospective studies [16,30,32,33,46,47]. Sixteen studies were performed as single-centre studies, and two were multi-centre studies [30,44]. The PRISMA flow diagram outlining the selection process is depicted in Appendix B. The reported mean age ranged from 1.5 to 5.0 years (mean = 2.8, CI 2.3, 3.3, I^2^ = 90.4%), and 218 (40.4%) patients were female. The pooled baseline characteristics of the included studies are described in Table 1.

### 3.2. Risk of Bias and Quality Assessment

The quality assessment of all the included studies is shown in Appendix C and Appendix D. The RCTs were evaluated via the RoB-2 Tool and were found to be of low risk [29,38,41], some concern [37,42] or high risk [34] of bias. Similarly, all the observational studies were assessed via the ROBINS-I Tool and were found to be of low [47], moderate [16,31,35,36,44,46] or serious [30,32,33,43,45] risk of bias. Those studies found to be of serious risk were due to lacking features in the domains of confounding, selection of participants, missing data, or selection of the reported results. Nonetheless, the patients appeared to represent the whole experience of the investigator, and all included studies adequately reported the selected exposure domains.

### 3.3. Clinical Characteristics

Among fifteen studies [16,29,31,32,33,34,35,36,38,41,42,43,44,45,46], 466 (85.8%) patients had scald burns, 51 (9.4%) with contact burns, 17 (3.1%) with flame burns, 3 (0.6%) with friction burns, 1 (0.2%) with radiation burns, and 5 (0.9%) with burns of other causes. Similarly, 415 (85.4%) patients had superficial partial thickness burns, 63 (13.0%) with deep partial thickness burns, 6 (1.2%) with full thickness burns, and 2 (0.4%) with superficial burns. Among nine studies [29,34,36,38,41,44,46,47] describing the anatomical site of burns, 142 (30.7%) were located in the trunk, 70 (15.1%) in the arm, forearm or wrist, 63 (13.6%) in the hand, 55 (11.9%) in the thigh, 33 (7.1%) in the foot, 21 (4.5%) in the face or neck, and 1 (0.2%) in the genital, perineal, or buttock area. Additionally, 78 (16.8%) patients had mixed burns. The pooled mean TBSA of burns prior to treatment among eleven studies was 5.2% (95% CI: 4.106, 6.269; I^2^ = 97.26%) [16,29,30,31,32,33,34,36,37,41,46]. The data regarding the clinical characteristics are summarised in Table 2.

### 3.4. Peri-Interventional Outcomes

**Figure 1:**
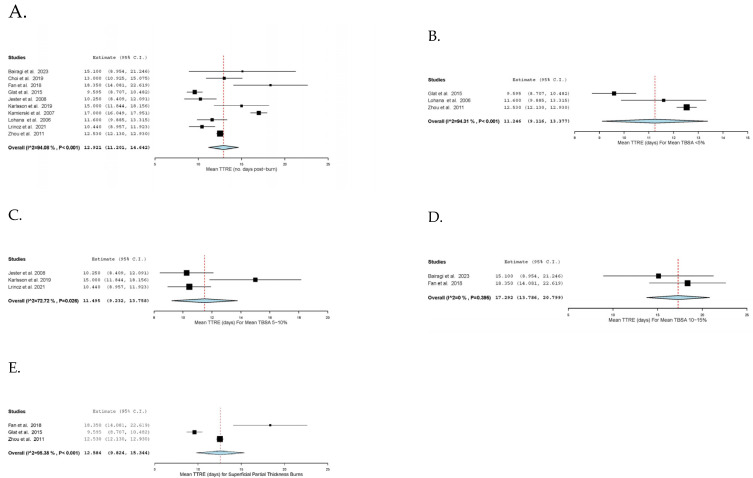
Mean time to re-epithelisation. (**A**). Mean time to re-epithelisation (days) for all burns. (**B**). Mean time to re-epithelisation (days) for burns with mean total body surface area between 0 and 5%. (**C**). Mean time to re-epithelisation (days) for burns with mean total body surface area between 5 and 10%. (**D**). Mean time to re-epithelisation (days) for burns with mean total body surface area between 10 and 15%. (**E**). Mean time to re-epithelization (days) for superficial partial thickness burns.

**Figure 2:**
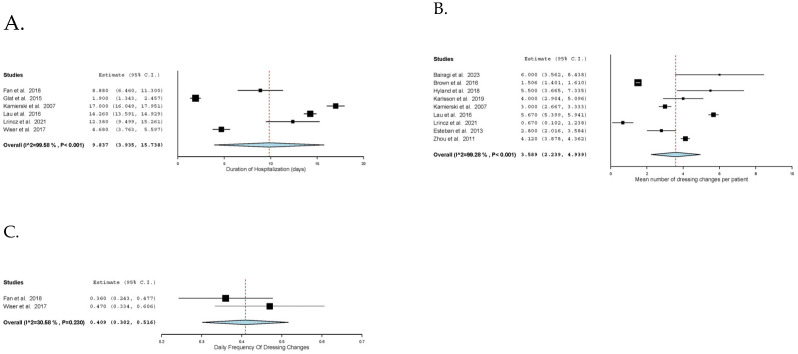
Features of clinical management. (**A**). Duration of hospitalisation (days). (**B**). Mean number of dressing changes per patient. (**C**). Daily frequency of dressing changes.

TTRE was reported in ten studies [29,30,31,32,33,34,35,36,37,38], and the overall pooled mean TTRE was 12.9 days (95% CI: 11.2, 14.6; I^2^ = 94%, *n* = 297). Additionally, a subgroup analysis on TTRE was performed based on the reported mean TBSA of the included studies. The pooled mean TTRE was 11.2 days (95% CI: 9.1, 13.4; I^2^ = 94%) for TBSA less than 5%, 11.5 days (95% CI: 9.2, 13.8; I^2^ = 73%) for TBSA between 5 and 10%, and 17.3 days (95% CI: 13.8, 20.8; I^2^ = 0%) for TBSA between 10 and 15%. Three studies solely analysed superficial partial thickness burns, and the pooled mean TTRE was 12.6 days (95% CI: 9.8, 15.3; I^2^ = 95%) [32,33,37]. The pooled mean hospital stays among six studies [16,32,33,34,36,46] was 9.8 days (95% CI: 3.9, 15.7; I^2^ = 100%). Among nine studies [16,29,34,35,36,37,41,42,45], the pooled mean number of dressing changes per patient was 3.6 times (95% CI: 2.2, 4.9; I^2^ = 99%). Similarly, the pooled mean daily frequency of dressing change was 0.4 times per day (95% CI: 0.3, 0.5; I^2^ = 31%), as reported in two studies [32,46]. Peri-interventional outcomes are comprehensively illustrated in Figure 1 and Figure 2.

### 3.5. Complications

Complications associated with the burn and dressing were reported in 17 studies [16,29,30,32,33,34,35,37,38,41,42,43,44,45,46,47]. A total of 98 complications were reported in the included studies, consisting of 30 (30.6%) infections, 29 (29.6%) requiring surgical intervention including skin grafting, 14 (14.3%) hypertrophic scarring, 17 (17.3%) escalations of care including unplanned representations or readmissions, 5 (5.1%) burn depth progression, and 3 (3.1%) others being sepsis, varicose veins, and allergic reaction.

**Figure 3:**
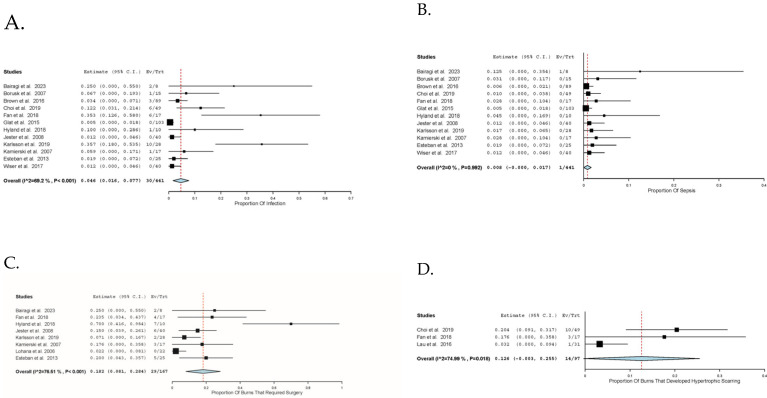
Proportion of complications. (**A**). Proportion of infections (%). (**B**). Proportion of sepsis (%). (**C**). Proportion of burns that required surgery (%). (**D**). Proportion of burns that developed hypertrophic scarring (%).

The proportion of patients who experienced wound infection was 4.6% (95% CI: 1.6, 7.7; I^2^ = 63%; *n* = 30), while 0.8% (95% CI: 0, 1.7; I^2^ = 0%; *n* = 1) of patients experienced sepsis in twelve studies [29,30,32,33,34,35,38,41,42,43,45,46]. The proportion of patients that required surgery or skin grafting after silver foam dressing was 18.2% (95% CI: 8.1, 28.4; I^2^ = 77%; *n* = 29) in eight studies [29,30,31,32,34,35,42,45]. This was divided into 13 (21.0%) out of 62 superficial partial-thickness burns in four studies [29,31,32,42,45], two (11.7%) out of 17 deep partial thickness in two studies [31,45] and three (100%) out of three third-degree burns in one study [45]. Eleven grafts in 85 patients were from three studies that did not report on their depths [30,34,35]. The proportion of patients that develop hypertrophic scarring was 12.6% (95% CI: 0.3, 25.5; I^2^ = 75% *n* = 14) in three studies [16,32,38]. Complications outcomes are comprehensively illustrated in Figure 3.

## 4. Discussion

Silver foam dressings are frequently used to manage paediatric burn injuries given their established antimicrobial properties [48]. However, debate exists on the healing rate and increased pain associated with frequent dressing changes in children. To the author’s knowledge, this is the first meta-analysis to demonstrate that the application of silver foam dressings in paediatric superficial to full-thickness burn injuries would result in (1) short re-epithelisation time, (2) low dressing change frequency, and (3) low wound infection rate. Although degrees of burn injury and anatomical location of burn injuries are likely variable and hard to generalise, our meta-analysis demonstrated that silver foam dressing is safe and effective and should be applied in clinical practice to achieve optimal outcomes for selected paediatric burn injuries.

Our meta-analysis demonstrated the pooled mean TTRE as 12.9 days, which was within the acceptable time frame of two weeks and consistent with existing meta-analyses among adult patients reporting a typical range of 10.2–17.9 days [49,50,51,52,53]. This was reassuring, given the concerns with the use of silver in children [15,54,55,56]. Importantly, however, this should be interpreted in the context of burn depth and TBSA, which have a significant influence on healing time. This was tentatively supported in this study where in burns with a mean TBSA < 5%, 5–10%, and 10–15%, the mean TTRE was 11.2, 11.5, and 17.3 days, respectively. A similar evaluation could not be made for burn depth due to lack of reporting; however, for superficial partial thickness burns alone, the mean TTRE was 12.58 days compared to 12.92 days in predominantly superficial partial thickness burns and deeper. Interestingly, two included studies found that, compared with a plain dressing product, silver improved healing, both noting the number of dressing changes to be lower in the silver foam dressing group compared to standard dressing [36,47]. Conversely [36,47], Fan et al. [32] found no difference in healing time and did not show any statistical difference between frequency of silver foam dressing and Biobrane. Nonetheless, further comparative studies with a control group after stratifying burn depth and TBSA are necessary to ascertain the effect of silver foam dressing on TTRE.

In this meta-analysis, paediatric burn was associated with a mean of 3.6 dressing changes, which was comparable to less than 3 dressing changes in a meta-analysis evaluating Acticoat, Mepitel, and Aquacel Ag in adults [50,53]. This supports the study by Lőrincz et al. [50] that the prolonged and sustained release of different silver foam dressings is comparable in terms of dressing frequency and less likely to affect the healing [57,58]. In addition to reduced costs and labour, this is also beneficial as fewer dressing changes reduce the risk of nosocomial infection and avoid unnecessary pain during changes, which can lead to non-compliance in children, hinder healing processes by disrupting the wound, and increase stress and inflammation [59,60,61]. Therefore, silver foam dressing changes are not performed more than necessary and are balanced with assessment and antimicrobial needs.

Our study demonstrated the use of silver foam dressings to be associated with infection rates of 4.6% in paediatric burns, within the previously reported range of 3.5–27.8% in adults [50,62]. Notably, positive infection swab cultures reported within included studies Karlsson et al. [34] and Selvarajah et al. [47] were especially high. The Karlsson study, which reported the highest bacterial load of the three at 86% of all swabs, admitted burns within 72 h of injury, while the other studies that reported mean time from injury to presentation were all less than 24 h. This supports that time to coverage has a significant impact on infection rates [32]. In looking at direct comparisons between dressing types, Selvarajah et al. [47] reported more than double the quantity of heavy growth swabs in the Biobrane group compared to the Acticoat group, noting also a higher dressing frequency in Biobrane. However, Lau et al. [16] reported the bacterial burden and infection rates to be comparable between silver and non-silver dressings but also reported significantly fewer dressing changes in silver foam dressings. Further studies are needed to characterise the trend of infective risk and antimicrobial coverage against the dressing frequency to determine the duration of silver dressing and inform the optimal time to re-introduce non-silver dressing and optimise cost.

As with all meta-analyses, limitations exist with this current study. First is the heterogeneity between studies of the intervention delivery (including dose, formulation, dressing frequency, interface of silver dressing, and co-interventions like saline and antiseptics) and the outcome measurement (including the healing start point and determination of healing), which limits the ability to draw discrete conclusions. Secondly, a lack of adequate reporting of common outcomes such as standard deviation of means, TBSA, burn depth, anatomical location, and rate of infection was a significant source of reporting bias, which limited the strength of the results and the ability to create subgroups to analyse data. This was crucial as TBSA, burn depth, and location have a significant impact on healing and frequency of dressing changes. Thirdly, overlap with patient complication datasets prevented determination of an overall complication rate within this meta-analysis. Fourthly, while it was a conscious decision to include single- or dual-arm studies about silver foam dressing to maximise the amount of data, a major drawback is the lack of comparative data from alternative dressings without silver, which prevented a two-arm analysis. Lastly, it is vital to recognise that these studies were performed predominantly on healthy children. However, outcomes of silver foam dressing are significantly affected by common paediatric comorbidities, such as diabetes, malnutrition, and obesity, which delay wound healing, increase infection risk, increase pressure on the wound site, and increase exudate production and dressing maintenance. Future comparative studies that clearly define and report healing outcomes (especially healing endpoint, TBSA, and burn depth) and include common paediatric comorbidities such as diabetes and obesity are necessary to (1) properly compare and assess the efficacy and safety of silver dressings, (2) investigate the possible heterogeneity in these outcomes and validate any findings, (3) enable more precise subgroups for these analysis, and (4) allow more targeted recommendations of the optimal dressing rate and duration to minimise toxicity and scarring while maximising the antimicrobial and re-epithelisation benefits of silver foam dressing.

## 5. Conclusions

This single-arm meta-analysis is the most comprehensive and current study to demonstrate the safety and efficacy of silver foam dressing use in selected paediatric burns. Notably, our study suggests low rates of mean TTRE, dressing frequency, and infection rates in paediatric patients, establishing a necessary benchmark for the use of silver foam dressings as reliable controls in future studies of emerging burn treatments. Beyond these results, further studies carefully consider crucial healing factors such as burn depth, TBSA, and underlying comorbidities to provide more targeted guidelines for the judicious use of silver foam dressings in paediatric burn management.

## Figures and Tables

**Table 1:** Baseline characteristics.

Study	Publication Year	Study Type	Country	Total Number of Participants (*n*)	Gender (Male/ Female)	Age, Mean ± SD (Years)
Bairagi et al. [29]	2023	RCT	Australia	8	4/4	1.5 ± 1.30
Borusk et al. [43]	2007	Prospective Cohort	Canada	15	7/8	5.5 ± NR
Brown et al. [41]	2015	RCT	NZ	89	49/40	3.6573 ± 3.80
Budkevich et al. [44]	2020	Prospective Cohort	Russia	94	60/34	2.7479 ± 3.22
Choi et al. [38]	2018	RCT	USA	49	34/15	1.58 ± 1.42
Fan et al. [32]	2018	Retrospective Study	Singapore	17	11/6	3.13 ± 3.14
Glat et al. [33]	2015	Retrospective Study	USA	103	NR/NR	4.4183 ± 4.13
Hyland et al. [42]	2018	RCT	Australia	10	8/2	1.5 ± NR
Jester et al. [30]	2008	Retrospective Study	UK	40	NR/NR	1.5 ± 3.51
Karlsson et al. [34]	2019	RCT	Sweden	28	16/12	1.427 ± 1.08
Kaźmierski et al. [35]	2007	Prospective Cohort	Poland	17	NR/NR	NR ± NR
Lau et al. [16]	2016	Retrospective Study	Hong Kong	31	17/14	2.38 ± 0.31
Lohana and Potokar [31]	2006	Prospective Cohort	UK	22	13/9	2.7 ± 1.88
Lőrincz et al. [36]	2021	Prospective Cohort	Hungary	9	6/3	4.88 ± 4.38
Paredes Esteban et al. [45]	2013	Prospective Cohort	Spain	25	18/7	4.2 ± 2.54
Selvarajah et al. [47]	2019	Retrospective Study	Australia	64	34/30	3.4 ± NR
Wiser et al. [46]	2017	Retrospective Study	Israel	40	24/16	2.9 ± 3.5
Zhou et al. [37]	2011	RCT	China	40	22/18	4.5 ± 2.20

*n*: number, NR: not reported, SD: standard deviation.

**Table 2:** Clinical characteristics.

Study	Publication Year	Burn Cause	Burn Depth	Burn Location	TBSA Mean ± SD
Bairagi et al. [29]	2023	Scalds (*n* = 7) Contact (*n* = 1) Flame (*n* = 0)	Superficial burns (*n* = 2) Superficial PTB (*n* = 8) Deep PTB (*n* = 5)	Face &/Neck (*n* = 8) Arm, forearm, wrist (*n* = 5) Hand (*n* = 3) Thigh, Leg (*n* = 5) Foot (*n* = 8) Trunk (*n* = 8)	11.50 ± 6.48
Borusk et al. [43]	2007	Scalds (*n* = 12) Contact (*n* = 1) Flame (*n* = 2)	Superficial PTB (*n* = 11) Deep PTB (*n* = 4)	NR	8.00 ± NR
Brown et al. [41]	2015	Scalds (*n* = 83) Contact (*n* = 6)	Superficial PTB (*n* = 89)	Face &/Neck (*n* = 2) Arm, forearm, wrist (*n* = 13) Thigh, Leg (*n* = 22) Trunk (*n* = 23) Mixed (*n* = 29)	2.45 ± 1.54
Budkevich et al. [44]	2020	Scalds (*n* = 93)	NR	Face &/Neck (*n* = 2) Arm, forearm, wrist (*n* = 20) Thigh, Leg (*n* = 11) Foot (*n* = 14) Mixed (*n* = 47)	43.11 ± 36.65
Choi et al. [38]	2018	Scalds (*n* = 5) Contact (*n* = 37) Flame (*n* = 4) Electrical (*n* = 0) Friction (*n* = 3)	Superficial PTB (*n* = 37) Deep PTB (*n* = 20) Full thickness burn (*n* = 3)	Arm, forearm, wrist (*n* = 2) Hand (*n* = 53) Thigh, Leg (*n* = 0) Foot (*n* = 5)	NR ± NR
Fan et al. [32]	2018	Scalds (*n* = 17) Flame (*n* = 0)	Superficial PTB (*n* = 17)	NR	11.68 ± 4.67
Glat et al. [33]	2015	Scalds (*n* = 95) Contact (*n* = 2) Flame (*n* = 4) Other (*n* = 2)	Superficial PTB (*n* = 103)	NR	3.26 ± 2.71
Hyland et al. [42]	2018	Scalds (*n* = 10)	NR	NR	8.50 ± NR
Jester et al. [30]	2008	NR	NR	NR	5.50 ± 5.57
Karlsson et al. [34]	2019	Scalds (*n* = 28)	NR	Face &/Neck (*n* = 0) Arm, forearm, wrist (*n* = 16) Thigh, Leg (*n* = 5) Trunk (*n* = 25)	5.00 ± 2.96
Kaźmierski et al. [35]	2007	Scalds (*n* = 17)	Superficial PTB (*n* = 17)	NR	NR ± NR
Lau et al. [16]	2016	Scalds (*n* = 30) Contact (*n* = 0) Flame (*n* = 1)	NR	NR	5.65 ± 0.58
Lohana and Potokar [31]	2006	Scalds (*n* = 15) Contact (*n* = 1) Flame (*n* = 2) Radiation (*n* = 1) Other (*n* = 3)	Superficial PTB (*n* = 17) Deep PTB (*n* = 5)	NR	4.00 ± 2.00
Lőrincz et al. [36]	2021	Scalds (*n* = 4) Contact (*n* = 2) Flame (*n* = 3)	Superficial PTB (*n* = 9)	Foot (*n* = 2) Mixed (*n* = 2)	5.27 ± 2.64
Paredes Esteban et al. [45]	2013	Scalds (*n* = 12)	Superficial PTB (*n* = 10) Deep PTB (*n* = 12) Full thickness burn (*n* = 3)	NR	NR ± NR
Selvarajah et al. [47]	2019	NR	Superficial PTB (*n* = 64)	Trunk (*n* = 64)	NR ± NR
Wiser et al. [46]	2017	Scalds (*n* = 38) Contact (*n* = 1) Flame (*n* = 1)	NR	Face &/Neck (*n* = 9) Arm, forearm, wrist (*n* = 14) Hand (*n* = 7) Thigh, Leg (*n* = 12) Foot (*n* = 4) Trunk (*n* = 22) Genitalia/perineum/buttock (*n* = 1)	4.90 ± 3.50
Zhou et al. [37]	2011	NR	Superficial burn (*n* = 0) Superficial PTB (*n* = 40) Full thickness burn (*n* = 0)	NR	3.85 ± 1.27

PTB: Partial Thickness Burn, NR: Not reported, SD: Standard Deviation.

## Data Availability

The original contributions presented in this study are included in the article. Further inquiries can be directed to the corresponding author(s).

## Appendix A. Search Strategy

ff. PubMed 135

((((Silver foam dressing) OR (““silver dressing””[Title/Abstract:~3])) OR (Mepilex Ag)) OR (Mepitel Ag)) OR (““silver dressings””[Title/Abstract:~3])

((““silver””[MeSH Terms] OR ““silver””[All Fields] OR ““silvers””[All Fields] OR ““silvered””[All Fields]) AND ““foam””[All Fields] AND (““bandages””[MeSH Terms] OR ““bandages””[All Fields] OR ““dressing””[All Fields] OR ““dressings””[All Fields] OR ““dress””[All Fields] OR ““dressed””[All Fields] OR ““dresses””[All Fields] OR ““dressing s””[All Fields])) OR ““silver dressing””[Title/Abstract:~3] OR (““Mepilex””[All Fields] AND (““agonists””[MeSH Subheading] OR ““agonists””[All Fields] OR ““ag””[All Fields])) OR ((““mepitel””[Supplementary Concept] OR ““mepitel””[All Fields]) AND (““agonists””[MeSH Subheading] OR ““agonists””[All Fields] OR ““ag””[All Fields])) OR ““silver dressings””[Title/Abstract:~3]

AND

((burns) OR (““burn””)) OR (scald)

““burning””[All Fields] OR ““burns””[MeSH Terms] OR ““burns””[All Fields] OR ““burned””[All Fields] OR ““burnings””[All Fields] OR ““burn””[All Fields] OR ““scald””[All Fields] OR ““scalded””[All Fields] OR ““scalding””[All Fields] OR ““scaldings””[All Fields] OR ““scalds””[All Fields]

AND

Infan* OR newborn* OR new-born* OR perinat* OR neonat* OR baby OR baby* OR babies OR toddler* OR ““minor”” OR minors* OR ““boy”” OR ““boys”” OR boyfriend OR boyhood OR girl* OR kid OR kids OR child OR child* OR children* OR schoolchild* OR schoolchild OR school child [tiab] OR school child*[tiab] OR adolescen* OR juvenil* OR youth* OR teen* OR underage* OR ““under age”” OR ““under aged”” OR pubescen* OR pediatrics[mh] OR pediatric* OR paediatric* OR peadiatric* OR prematur* OR preterm*

““infan*””[All Fields] OR ““newborn*””[All Fields] OR ““new born*””[All Fields] OR ““perinat*””[All Fields] OR ““neonat*””[All Fields] OR (““infant, newborn””[MeSH Terms] OR (““infant””[All Fields] AND ““newborn””[All Fields]) OR ““newborn infant””[All Fields] OR ““baby””[All Fields] OR ““infant””[MeSH Terms] OR ““infant””[All Fields]) OR ““baby*””[All Fields] OR (““baby s””[All Fields] OR ““babys””[All Fields] OR ““infant””[MeSH Terms] OR ““infant””[All Fields] OR ““babies””[All Fields]) OR ““toddler*””[All Fields] OR ““minor””[All Fields] OR ““minors*””[All Fields] OR ““boy””[All Fields] OR ““boys””[All Fields] OR (““boyfriend””[All Fields] OR ““boyfriend s””[All Fields] OR ““boyfriends””[All Fields]) OR ““boyhood””[All Fields] OR ““girl*””[All Fields] OR ““kid””[All Fields] OR ““kids””[All Fields] OR (““child””[MeSH Terms] OR ““child””[All Fields] OR ““children””[All Fields] OR ““child s””[All Fields] OR ““children s””[All Fields] OR ““childrens””[All Fields] OR ““childs””[All Fields]) OR ““child*””[All Fields] OR ““children*””[All Fields] OR ““schoolchild*””[All Fields] OR ““schoolchild””[All Fields] OR ““school child””[Title/Abstract] OR ““school child*””[Title/Abstract] OR ““adolescen*””[All Fields] OR ““juvenil*””[All Fields] OR ““youth*””[All Fields] OR ““teen*””[All Fields] OR ““underage*””[All Fields] OR ““under age””[All Fields] OR ““under aged””[All Fields] OR ““pubescen*””[All Fields] OR ““pediatrics””[MeSH Terms] OR ““pediatric*””[All Fields] OR ““paediatric*””[All Fields] OR ““peadiatric*””[All Fields] OR ““prematur*””[All Fields] OR ““preterm*””[All Fields]

EMBASE 226

Embase

Session Results

.......................................................

No. Query Results Results Date

#18. #5 AND #10 AND #17 226 9 Oct 2023

#17. #11 OR #12 OR #13 OR #14 OR #15 OR #16 7,009,376 9 Oct 2023

#16. infan* OR newborn* OR ’new born*’’ OR perinat* OR 7,009,376 9 Oct 2023

neonat* OR baby OR baby* OR babies OR toddler* OR

’minor’ OR minors* OR ’boy’ OR ’boys’ OR

boyfriend OR boyhood OR girl* OR kid OR kids OR

child OR child* OR children* OR schoolchild* OR

schoolchild OR adolescen* OR juvenil* OR youth*

OR teen* OR underage* OR ’under age’ OR ’under

aged’ OR pubescen* OR pediatrics OR pediatric* OR

paediatric* OR peadiatric* OR prematur* OR

preterm*

#15. ’pediatrics’/exp OR pediatrics 1,061,695 9 Oct 2023

#14. ’baby’/exp OR baby 84,109 9 Oct 2023

#13. ’infant’/exp OR infant 1,409,307 9 Oct 2023

#12. ’adolescent’/exp OR adolescent 2,067,036 9 Oct 2023

#11. ’child’/exp OR child 3,901,737 9 Oct 2023

#10. #6 OR #7 OR #8 OR #9 368,420 9 Oct 2023

#9. scald* 8,675 9 Oct 2023

#8. ’scald’/exp OR scald 4,216 9 Oct 2023

#7. burn* 359,086 9 Oct 2023

#6. ’burn’/exp OR burn 128,398 9 Oct 2023

#5. #1 OR #2 OR #3 OR #4 2,043 9 Oct 2023

#4. silver NEAR/3 dressing* 1,795 9 Oct 2023

#3. ’mepitel ag’ 6 9 Oct 2023

#2. ’mepilex ag’ 81 9 Oct 2023

#1. ’silver foam dressing’ OR ((’silver’/exp OR 531 9 Oct 2023

silver) AND (’foam’/exp OR foam) AND

(’dressing’/exp OR dressing))

.......................................................

Cochrane 66

Search Name:

Date Run: 10/10/2023 09:06:03

Comment:

ID Search  Hits

#1 (Silver foam dressing):ti,ab,kw (Word variations have been searched)  81

#2 (Silver NEAR/3 dressing*):ti,ab,kw (Word variations have been searched)  428

#3 (Mepilex Ag):ti,ab,kw (Word variations have been searched)  30

#4 (Mepitel Ag):ti,ab,kw (Word variations have been searched)  10

#5 {OR #1-#4  458

#6 MeSH descriptor: [Burns] explode all trees  2134

#7 (burn):ti,ab,kw (Word variations have been searched)  11,380

#8 (scald):ti,ab,kw (Word variations have been searched)  164

#9 #6 OR #7 OR #8  11,670

#10 MeSH descriptor: [Child] explode all trees  78,615

#11 MeSH descriptor: [Adolescent] explode all trees  125,922

#12 MeSH descriptor: [Infant] explode all trees  42,054

#13 (Infant OR Infan* OR newborn* OR new-born* OR perinat* OR neonat* OR baby OR baby* OR babies OR toddler* OR ““minor”” OR minors* OR ““boy”” OR ““boys”” OR boyfriend OR boyhood OR girl* OR kid OR kids OR child OR child* OR children* OR schoolchild* OR schoolchild OR school child OR school child* OR adolescen* OR juvenil* OR youth* OR teen* OR underage* OR “under age” OR “under aged” OR pubescen* OR pediatrics OR pediatric* OR paediatric* OR peadiatric* OR prematur* OR preterm*):ti,ab,kw (Word variations have been searched)  388,895

#14 {OR #10-#13}  388,895

#15 #5 AND #9 AND #14  66

CiNAHL 72

**#**	**Query**	**Limiters/Expanders**	**Last Run Via**	**Results**
S15	S5 AND S9 AND S14	Expanders—Apply equivalent subjects Search modes—Boolean/Phrase	Interface—EBSCOhost Research Databases Search Screen—Advanced Search Database—CINAHL Complete	72
S14	S10 OR S11 OR S12 OR S13	Expanders—Apply equivalent subjects Search modes—Boolean/Phrase	Interface—EBSCOhost Research Databases Search Screen—Advanced Search Database—CINAHL Complete	1,602,907
S13	infan* OR newborn* OR ’new born*’’ OR perinat* OR neonat* OR baby OR baby* OR babies OR toddler* OR ’minor’ OR minors* OR ’boy’ OR ’boys’ OR boyfriend OR boyhood OR girl* OR kid OR kids OR child OR child* OR children* OR schoolchild* OR schoolchild OR adolescen* OR juvenil* OR youth* OR teen* OR underage* OR ’under age’ OR ’u’nder aged’ OR pubescen* OR pediatrics OR pediatric* OR paediatric* OR peadiatric* OR prematur* OR preterm*	Expanders—Apply equivalent subjects Search modes—Boolean/Phrase	Interface—EBSCOhost Research Databases Search Screen—Advanced Search Database—CINAHL Complete	1,602,907
S12	(MM ““Pediatrics+””)	Expanders—Apply equivalent subjects Search modes—Boolean/Phrase	Interface—EBSCOhost Research Databases Search Screen—Advanced Search Database—CINAHL Complete	11,582
S11	(MM ““Infant+””)	Expanders—Apply equivalent subjects Search modes—Boolean/Phrase	Interface—EBSCOhost Research Databases Search Screen—Advanced Search Database—CINAHL Complete	24,769
S10	(MM ““Child+””)	Expanders—Apply equivalent subjects Search modes—Boolean/Phrase	Interface—EBSCOhost Research Databases Search Screen—Advanced Search Database—CINAHL Complete	43,831
S9	S6 OR S7 OR S8	Expanders—Apply equivalent subjects Search modes—Boolean/Phrase	Interface—EBSCOhost Research Databases Search Screen—Advanced Search Database—CINAHL Complete	70,480
S8	scald*	Expanders—Apply equivalent subjects Search modes—Boolean/Phrase	Interface—EBSCOhost Research Databases Search Screen—Advanced Search Database—CINAHL Complete	1309
S7	burn*	Expanders—Apply equivalent subjects Search modes—Boolean/Phrase	Interface—EBSCOhost Research Databases Search Screen—Advanced Search Database—CINAHL Complete	69,991
S6	(MM ““Burns+””)	Expanders—Apply equivalent subjects Search modes—Boolean/Phrase	Interface—EBSCOhost Research Databases Search Screen—Advanced Search Database—CINAHL Complete	17,224
S5	S1 OR S2 OR S3 OR S4	Expanders—Apply equivalent subjects Search modes—Boolean/Phrase	Interface—EBSCOhost Research Databases Search Screen—Advanced Search Database—CINAHL Complete	941
S4	silver N3 dressing*	Expanders—Apply equivalent subjects Search modes—Boolean/Phrase	Interface—EBSCOhost Research Databases Search Screen—Advanced Search Database—CINAHL Complete	929
S3	mepitel ag	Expanders—Apply equivalent subjects Search modes—Boolean/Phrase	Interface—EBSCOhost Research Databases Search Screen—Advanced Search Database—CINAHL Complete	3
S2	mepilex ag	Expanders—Apply equivalent subjects Search modes—Boolean/Phrase	Interface—EBSCOhost Research Databases Search Screen—Advanced Search Database—CINAHL Complete	28
S1	silver foam dressing	Expanders—Apply equivalent subjects Search modes—Boolean/Phrase	Interface—EBSCOhost Research Databases Search Screen—Advanced Search Database—CINAHL Complete	94

Scopus 158

((TITLE-ABS-KEY(silver foam dressing)) OR (TITLE-ABS-KEY(mepilex ag)) OR (TITLE-ABS-KEY(mepitel ag)) OR (TITLE-ABS-KEY(silver W/3 dressing*)))

AND (TITLE-ABS-KEY(burn* OR scald*))

AND (TITLE-ABS-KEY(infan* OR newborn* OR “new born*” OR perinat* OR neonat* OR baby OR baby* OR babies OR toddler* OR “minor” OR minors* OR “boy” OR “boys” OR boyfriend OR boyhood OR girl* OR kid OR kids OR child OR child* OR children* OR schoolchild* OR schoolchild OR adolescen* OR juvenil* OR youth* OR teen* OR underage* OR “under age” OR “under aged” OR pubescen* OR pediatrics OR pediatric* OR paediatric* OR peadiatric* OR prematur* OR preterm*))

Web of Science 76

# Web of Science Search Strategy (v0.1)

# Database: Web of Science Core Collection

# Entitlements:

- WOS.IC: 1993 to 2023

- WOS.CCR: 1985 to 2023

- WOS.SCI: 1900 to 2023

- WOS.AHCI: 1975 to 2023

- WOS.BHCI: 2005 to 2023

- WOS.BSCI: 2005 to 2023

- WOS.ESCI: 2005 to 2023

- WOS.ISTP: 1990 to 2023

- WOS.SSCI: 1900 to 2023

- WOS.ISSHP: 1990 to 2023

# Searches:

1: TS=(silver foam dressing)      Date Run: Thu Oct 12 2023 06:59:28 GMT+1000 (Australian Eastern Standard Time)   Results: 197

2: TS=(mepilex ag)      Date Run: Thu Oct 12 2023 07:00:05 GMT+1000 (Australian Eastern Standard Time)   Results: 24

3: TS=(mepitel ag)      Date Run: Thu Oct 12 2023 07:00:41 GMT+1000 (Australian Eastern Standard Time)   Results: 7

4: TS=(silver NEAR/3 dressing*)      Date Run: Thu Oct 12 2023 07:06:28 GMT+1000 (Australian Eastern Standard Time)   Results: 1236

5: #1 OR #2 OR #3 OR #4      Date Run: Thu Oct 12 2023 07:06:55 GMT+1000 (Australian Eastern Standard Time)   Results: 1299

6: TS=(burn* OR scald*)      Date Run: Thu Oct 12 2023 07:07:35 GMT+1000 (Australian Eastern Standard Time)   Results: 301,703

7: TS=(infan* OR newborn* OR ’n’ew born*’’ OR perinat* OR neonat* OR baby OR baby* OR babies OR toddler* OR ’m’inor’ OR minors* OR ’b’oy’ OR ’b’oys’ OR boyfriend OR boyhood OR girl* OR kid OR kids OR child OR child* OR children* OR schoolchild* OR schoolchild OR adolescen* OR juvenil* OR youth* OR teen* OR underage* OR ’u’nder age’ OR ’u’nder aged’ OR pubescen* OR pediatrics OR pediatric* OR paediatric* OR peadiatric* OR prematur* OR preterm*)      Date Run: Thu Oct 12 2023 07:08:11 GMT+1000 (Australian Eastern Standard Time)   Results: 4,865,845

8: #5 AND #6 AND #7      Date Run: Thu Oct 12 2023 07:08:42 GMT+1000 (Australian Eastern Standard Time)   Results: 76
